# Trends in Coronary Artery Anomalies Detection by Coronary Computed Tomography Angiography (CCTA): A Real-Life Comparative Study before and during the COVID-19 Pandemic

**DOI:** 10.3390/healthcare12111091

**Published:** 2024-05-26

**Authors:** Alexandra-Simona Zamfir, Tudor-Andrei Cernomaz, Bogdan Mihnea Ciuntu, Doina Azoicăi, Carmen Lăcrămioara Zamfir, Raluca Ozana Chistol, Anca Sava

**Affiliations:** 1 Clinical Hospital of Pulmonary Diseases, 700115 Iasi, Romania; simona-zamfir@umfiasi.ro; 2 Department of Medical Sciences III, Faculty of Medicine, “Grigore T. Popa” University of Medicine and Pharmacy, 700115 Iasi, Romania; 3 Regional Institute of Oncology, 700483 Iasi, Romania; 4 Department of Surgery, “Grigore T. Popa” University of Medicine and Pharmacy, 700115 Iasi, Romania; bogdan-mihnea.ciuntu@umfiasi.ro; 5 Department of Surgery, “St. Spiridon” County Clinical Emergency Hospital, 700111 Iasi, Romania; 6 Department of Preventive Medicine and Interdisciplinarity, “Grigore T. Popa” University of Medicine and Pharmacy, 700115 Iași, Romania; doina.azoicai@umfiasi.ro; 7 Department of Morpho-Functional Sciences I, Faculty of Medicine, “Grigore T. Popa” University of Medicine and Pharmacy, 700115 Iasi, Romania; carmen.zamfir@umfiasi.ro (C.L.Z.); raluca-ozana.chistol@umfiasi.ro (R.O.C.); sava.anca@umfiasi.ro (A.S.); 8 Department of Medical Imaging, “Prof. Dr. George I.M. Georgescu” Cardiovascular Diseases Institute, 700503 Iași, Romania

**Keywords:** COVID-19 pandemic, coronary artery anomalies, cardiovascular risks, public health

## Abstract

Background: In the wake of the coronavirus disease 19 (COVID-19) pandemic, affecting healthcare systems globally, urgent research is needed to understand its potential repercussions on the diagnosis and management of cardiovascular disorders. This emphasises the importance of detecting coronary artery anomalies (CAAs), rare conditions that can range from benign to potentially life-threatening manifestations. We aimed to retrospectively assess the impact of the COVID-19 pandemic on the detection of various coronary anomalies using Coronary Computed Tomography Angiography (CCTA) within a regional tertiary cardiology unit in north-eastern Romania, focusing on perceived occurrence in the population under study, types, and related demographic and clinical factors. Methods: We analysed CCTA scans and investigated the trends in CAA detection among cardiology patients over a decade. We compared pre-COVID-19 and pandemic-era data to assess the impact of healthcare utilisation, patient behaviour, and diagnostic approaches on anomaly detection. Results: Our analysis revealed a higher detection rate of CAAs during the pandemic (3.9% versus 2.2%), possibly highlighting differences in patient clinical profile and addressability changes presentation compared to the previous period. Origination and course anomalies, often linked to severe symptoms, were significantly higher pre-COVID-19 (64.1% versus 51.3%). Conversely, intrinsic CAAs, typically asymptomatic or manifesting later in life, notably increased during the pandemic (49.0% versus 61.4%; *p* = 0.020). Conclusions: Our study underscores a significant rise in CAA detection during the COVID-19 era, potentially linked to changes in cardiovascular and respiratory clinical patterns, with advanced imaging modalities like CCTA offering accuracy in identification.

## 1. Introduction

### 1.1. Background

Coronary artery anomalies (CAAs) are part of the broad spectrum of congenital disorders, which involve an abnormality of one of the three main epicardial coronary arteries, with an estimated prevalence of 1–2% in the general population [1,2]. Despite significant developments in cardiovascular imaging, CAAs remain insufficiently explored with limited data available on prevalence or clinical impact. These anomalies present as variations in the origin, trajectory, structure, or termination of the coronary arteries, often attributed to perturbations during embryological development [2,3]. They can range from benign and asymptomatic to presenting various symptoms, including chest pain, dyspnea, irregular heart rhythm, and, in extreme cases, sudden death [2]. Nevertheless, CAAs have been associated with physical activity-related arrhythmias and are ranked as the second most common cause of sudden death in young adults engaged in competitive sports [4,5]. This underscores the importance of thorough screening and evaluation for CAAs, even in cases where symptoms may not be initially present.

The taxonomy proposed by Angelini, widely accepted as the most comprehensive, consists of four distinct groups: anomalies of origination and course (Group A), anomalies of intrinsic coronary arterial anatomy (Group B), anomalies of coronary termination (Group C), and anomalous anastomotic vessels (Group D) [6]. Type A anomalies, which involve abnormal origin and course of coronary arteries, are often associated with insufficient myocardial perfusion, possibly followed by hypoxic events [7]. This category includes a range of anomalies, such as the absence of the left main trunk, abnormal positioning of the coronary ostium, or the presence of a single coronary artery [6]. At the same time, patients with these anomalies are more likely to be symptomatic with clinical manifestations such as angina, myocardial infarction, or sudden cardiac death [7]. Given that these anomalies may manifest first as sudden death, Angelini proposed the development of a screening protocol in 2007; still, there is no published data available regarding the successful implementation of such an approach [6]. Group B encompasses a spectrum of anomalies, ranging from congenital ostial stenosis and atresia to coronary ectasia, hypoplasia, muscular bridges, and aberrant origins of the posterior descending artery or first septal branch [6]. Anomalies ascribed to groups C and D are deemed rare, with few published relevant cases up to date.

In everyday clinical practice, diagnoses frequently arise incidentally during investigations prompted by other medical conditions. While formerly detected primarily through autopsy, nowadays advancements in cardiac imaging techniques have significantly improved and refined the precise identification of these anomalies [4]. Utilising Coronary Computed Tomography Angiography (CCTA) emerges as a highly effective strategy for detecting CAAs, providing precise identification and detailed imaging in a non-invasive manner. Its capability to accurately identify even the most severe and malignant anomalies enhances its value in clinical practice, facilitating the diagnosis and management of CAAs [8].

The World Health Organisation (WHO) declared the infection caused by the severe acute respiratory syndrome coronavirus 2 (SARS-CoV-2) a global pandemic in March 2020. This virus led to the novelty of coronavirus disease 19 (COVID-19), primarily recognised for respiratory symptoms, but also multiple other systemic complications [9].

The overlapping cardiometabolic profile observed in both COVID-19 and cardiac diseases suggests a potential role for COVID-19 in exacerbating subclinical conditions such as coronary artery disease and heart failure [10]. The pandemic impact meant not only a new disease, but also changing medical practices and protocols to conform to epidemiological restrictions and to tackle increased needs of healthcare. Therefore, it is a necessity to understand the shifts in the diagnosis and management of not only prevalent cardiovascular diseases, but also rare conditions like CAAs [2].

At the same time, we need to acknowledge how the manifestations of COVID could trigger or intersect with those of CAAs, within the wide spectrum of cardiovascular complications associated with the SARS-CoV-2 infection. However, while investigating this interference is essential, it is important to acknowledge potential challenges.

Variations in healthcare access, diagnostic protocols, and patient behaviours during the pandemic may complicate efforts to accurately assess the occurrence and manifestations of CAAs. Despite these challenges, continued research and awareness are necessary to address the evolving landscape of congenital CAAs amid the COVID-19 pandemic.

### 1.2. Aims

We aimed to retrospectively assess the impact of the COVID-19 pandemic on the detection of various coronary anomalies using CCTA within a regional tertiary cardiology unit in north-eastern Romania, focusing on perceived occurrence in the population under study, types, and related demographic and clinical factors. Through the comparative analysis of data from before and during the pandemic period, our objective was to determine whether shifts in healthcare utilisation, patient behaviour, or alterations in diagnostic practices influenced the detection of CAAs. Our study’s significance is underscored by the lack of research investigating the impact of COVID-19 on patients with CAAs.

## 2. Materials and Methods

### 2.1. Study Design and Patients

Medical data from patients referred for CCTA was retrospectively assessed and analysed. The investigated patients underwent CCTA within two epidemiologically defined timeframes: the period preceding the COVID-19 outbreak (2012–2019) and the years during the pandemic (2020–2022). Inclusion criteria referred to medical data sets originating from individuals aged 18 years and older, who provided informed consent for the utilisation of their medical data. The only exclusion criterion considered was the unavailability of imagistic data—there were no such cases. The following parameters were recorded: demographic data (such as gender and age) and the specific group type of anomalies diagnosed for each patient. Principal symptoms reported by patients, including angina, dyspnea, syncope, and fatigue, together with concurrent electrocardiogram (ECG) changes, were included. We evaluated the cardiovascular risk factors, encompassing hypertension, diabetes, dyslipidemia, metabolic syndrome, and smoking history, alongside the coronary artery calcium score (CAC), indicating calcification. Scores were categorised as absent, mild (1–100), moderate (101–400), or severe (above 400) based on established criteria. Furthermore, the presence of coronary artery disease (CAD), chronic cardiac failure, and dilated cardiomyopathy was assessed.

The CCTA scans were obtained from the database of “Prof. Dr. George I.M. Georgescu” Cardiovascular Diseases Institute from Iasi, Romania. Available CCTAs were recommended and performed according to local standard of practice: all patients had either known coronary heart disease or symptomatology compatible with such a diagnosis. Local guideline lists some particular conditions as contraindications for CCTA: pregnancy, severe renal failure, and hemodynamic instability. No data were available on how many patients were thus denied CCTA.

The imaging procedures were performed on a second-generation 128-slice dual-source CT scanner (Somatom Definition Flash; Siemens Healthcare, Forchheim, Germany). Images were acquired prior to and following the intravenous injection of 80–120 mL of iodinated contrast agent (Iomeron 400, Bracco, Milan, Italy), based on body weight using an infusion rate of 3.5–5 mL/s, followed by a saline chaser delivered at the same flow rate. Reconstruction of images at various percentage times of an R-R interval, with a slice thickness of 0.75 mm, was optimised and conducted. Subsequently, the reconstructed images were transferred to a Syngo.via workstation (Siemens Healthineers) for comprehensive image analysis. CCTA series were independently assessed by two experienced radiologists proficient in cardiac imaging, according to local diagnostic protocols. In instances of discrepancy, consensus was achieved through collaborative discussion.

Despite our careful application of selection criteria, it is important to acknowledge potential limitations in the retrospective design, which may introduce challenges such as variations in diagnostic practices over time. Additionally, reliance on existing medical records, imaging studies, and CCTA may have slightly constrained the breadth of available data for analysis.

The study design and protocol were approved by the Ethics Committee of “Grigore T. Popa” University of Medicine and Pharmacy Iasi, Romania (no. 115/15 October 2021).

### 2.2. Statistical Analysis

The statistical analysis was conducted using Statistical Package for Social Sciences v.27 (IBM Corp. Armonk, NY, USA). Differences were assessed using the chi squared test for categorical data and the two-tailed t-student for continuous variables (after checking for normality). All analyses were conducted within a significance level of *p* < 0.05.

## 3. Results

In our study, we identified 8733 CCTA scans conducted before the COVID-19 pandemic, covering the period from 2012 to 2019 (average 1091/annum), among which anomalies were detected in 192 patients (2.2%). Subsequently, over the ensuing three years of the COVID-19 era, 4025 CCTA scans were performed (average 1341/annum), identifying coronary anomalies in 158 patients (3.9%) among the population with symptoms, fulfilling the recommendations for CCTA.

**Figure 1:**
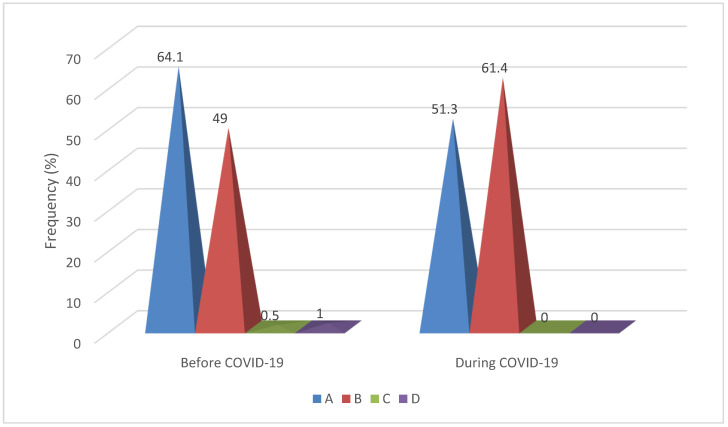
Coronary artery anomalies groups (A, B, C, D) distribution detected by CCTA before and during COVID-19.

A shift in dominance becomes evident when examining variations among the distinct groups of CAAs. The proportion of patients with group A anomalies of origination and course was significantly higher before the COVID-19 period (64.1% versus 51.3%; *p* = 0.016), while the proportion of group B anomalies of intrinsic anatomy was significantly higher during the pandemic (49.0% versus 61.4%; *p* = 0.020). The small number of cases with group C (n = 1) or D (n = 2) anomalies recorded only before the COVID-19 period does not allow the application of statistical significance tests (Figure 1).

**Figure 2:**
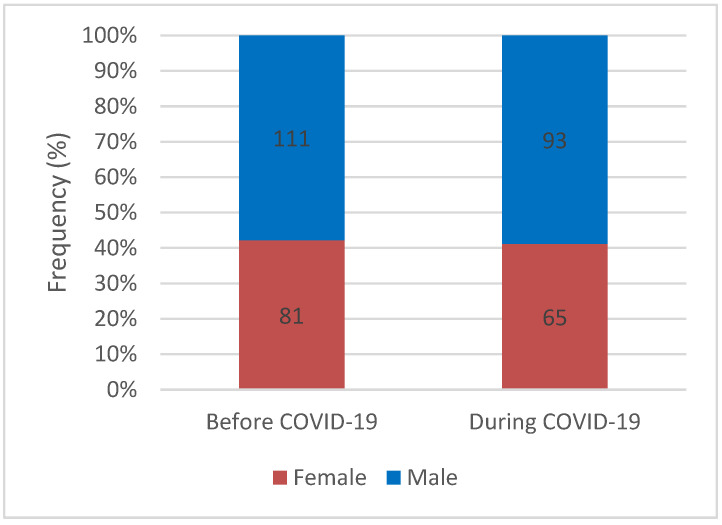
Gender distribution of coronary artery anomalies before and during the COVID-19 pandemic.

Despite considerable numerical variations among patients diagnosed with CAAs, gender distribution remained remarkably consistent across both evaluated time frames, with men exhibiting a higher occurrence of anomalies (57.8% versus 58.6%; *p* = 0.843) (Figure 2).

**Figure 3:**
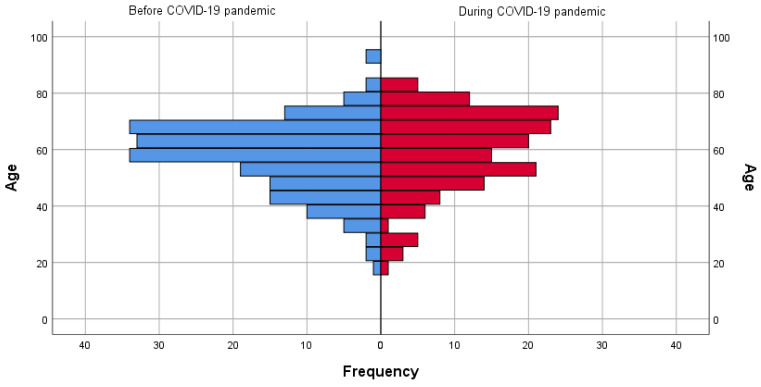
Population pyramid comparisons before and during the COVID-19 pandemic.

The population pyramid during and before the COVID-19 pandemic highlights subtle differences in age distribution between these timeframes (Figure 3). In the pre-COVID-19 era, the age range of individuals with CAAs varied from 18 to 93 years, while during the pandemic, it fluctuated between 20 and 84 years. The average age of patients before COVID-19 was slightly lower than that during COVID-19 (57.61 years ± 12.63 versus 59.55 years ± 14.19; *p* = 0.177, respectively).

The analysis of symptoms revealed that angina was the predominant complaint among patients (54.3%), followed by dyspnea (41.4%), fatigue (34.9%), palpitations (31.1%), and syncope (3.1%) (Table 1). Angina emerged as the primary presentation before COVID-19, although not statistically significant (*p* = 0.058). However, during COVID-19, dyspnea showed a significant increase in frequency compared to pre-pandemic levels (*p* = 0.037), along with fatigue (*p* = 0.029).

While observing the standard 12 lead resting ECG abnormalities during these two time frames, supraventricular tachycardia emerged as the most frequent and was particularly more prevalent before the COVID-19 era (Table 2). Notably, during this period, the most common abnormalities were ST-elevation myocardial infarction (STEMI) (*p* = 0.002), along with malignant ventricular dysrhythmias (*p* = 0.024) and right bundle branch block (*p* = 0.038). Although not statistically significant, during the COVID-19 era, there was an observed increase in the prevalence of atrioventricular blocks, left bundle branch blocks, premature atrial and ventricular complexes, as well as non-STEMI.

Furthermore, patients exhibited risk factors predisposing them to coronary artery disease (CAD), including hypertension, diabetes mellitus, dyslipidemia (collectively indicative of metabolic syndrome), or smoking. Although these factors are not directly linked to CAAs, they often prompt patients to seek medical attention and, in some situations, to undergo CCTA, thereby contributing to the detection of coronary anomalies (Table 3).

**Figure 4:**
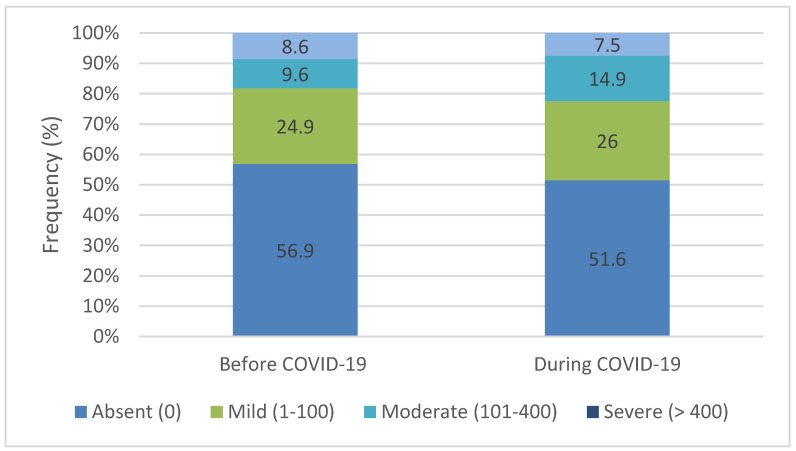
The structure of the study cohort according to the calcium score level (cardiovascular risk assessment).

In both study groups, cases with absent calcium scores predominated (56.9% versus 51.6%), while the risk of severe cardiovascular events was below 9% (8.6% versus 7.5%) (*p* = 0.416) (Figure 4).

In terms of the associated comorbidities of these patients, cardiovascular conditions remained prevalent across both selected time frames, with chronic cardiac failure exhibiting the highest prevalence, followed by coronary artery disease (CAD) and dilated cardiomyopathy (Table 4).

## 4. Discussion

Coronary Computed Tomography Angiography (CCTA), recognised for its specificity and accuracy, provides a non-invasive alternative for detecting CAAs, surpassing catheter angiography in diagnostic capability [11]. CCTA has become a key component in the cardiac care of patients with chronic coronary syndrome, particularly since it was designated as a first-line imaging modality for stable chest pain by the European Society of Cardiology in 2019. During the COVID-19 pandemic, there was a notable shift towards safer imaging procedures, leading to increased utilisation of CCTA as an alternative to invasive procedures like coronary angiography and transesophageal echocardiography. By implementing adapted protocols for thorough cardiopulmonary evaluation, CT scans can offer important insights into both cardiac and pulmonary conditions [12]. At the same time, it not only allows for the investigation of patients already known with chronic cardiac diseases, but also plays an important role in diagnosing an acute cardiac event in patients with COVID-19 pneumonia and increased troponin serum values. CCTA underscores its cost-effectiveness by facilitating swift and precise diagnosis of coronary artery disease, leading to shortened hospital stays and appropriate therapeutic interventions. Consequently, it might be considered a useful tool in reducing mortality associated with CAD [9].

Our data suggest a higher detection of the CAAs with CCTA during the COVID-19 era using 2012–2019 as a comparator, with figures of 4% versus 2%, respectively, in the context of similar numbers of scans per annum. There is no simple and clear-cut explanation for these figures and multiple hypotheses may be put forward. With prevalence rates anticipated to remain stable in the general population over several years, and no significant demographic shifts expected in the area served, alongside an unchanged referral/examination protocol, any surplus of diagnoses is likely a result of pandemic-related disruptions to the functioning of the public health system.

Public health measures may partially explain such results—a deliberate prioritisation of certain diagnostic procedures, such as CCTA, for patients with suspected or confirmed COVID-19 with cardiovascular concerns might have been carried out. This matter highlights the rising recognition of cardiac complications associated with the virus, which carry significant morbidity and mortality burdens [13]. This increased utilisation of CCTA could have led to the incidental detection of previously undiagnosed and asymptomatic CAAs. Additionally, the heightened awareness of health risks associated with COVID-19 might have prompted individuals to seek medical attention for symptoms they would have otherwise ignored. While lockdown measures may have influenced primary care presentations to some extent, it is important to note that during the COVID-19 pandemic, CT imaging, particularly for thoracic conditions, thrived. CT scans were considered the gold standard for diagnosing respiratory illnesses, including COVID-19 pneumonia [14]. Therefore, despite potential changes in primary care utilisation, the demand for thoracic imaging remained high, reflecting its importance in diagnosing respiratory conditions during the pandemic. This trend is illustrated by the figures reported by the Organization for Economic Cooperation and Development (OECD) concerning the number of CT scans per annum per 1000 inhabitants in Romania; available data show a significant increase during the COVID-19 era, rising from 34 in 2019 to 36.4 in 2020, and reaching 57.8 in 2021 [15]. Such a trend might explain the higher detection of CAAs in terms of more serendipitous findings following an increased number of performed CCTAs. This in turn might reflect COVID-related changes in healthcare utilisation possibly linked to heightened awareness of cardiovascular diseases and their implications.

During the COVID-19 era, the similarity in symptoms between CAAs and COVID-19 might have posed diagnostic difficulties. While COVID-19 predominantly revolves around respiratory symptoms, attributed to the lung’s susceptibility to SARS-CoV-2, which triggers interstitial pneumonia, the overlapping symptoms, including chest pain and irregular heart rhythms, might have led to challenges in diagnosis. Interestingly, the cardiovascular system emerges as a significant target for SARS-CoV-2, with cardiac injury strongly associated with adverse clinical consequences. Several studies have documented diverse cardiovascular manifestations in COVID-19 patients, spanning from myocarditis and acute coronary syndrome (ACS) to arrhythmias or thromboembolic events [16]. Nevertheless, our results showed that during the pandemic, the most frequent and relevant resting ECG abnormalities emphasised atrioventricular blocks, left bundle branch blocks, as well as non-STEMI. Accurate and timely discovery of CAAs enables healthcare providers to make informed decisions about additional diagnostic tests or treatment options, thereby managing potential complications and reducing the risk of adverse events. These complications may include spasm, atherosclerosis development, myocardial infarction, or sudden cardiac death. By optimising patient care through early detection, healthcare professionals can potentially alleviate the burden associated with these conditions [2,6].

In our study, significant statistical differences have been observed specifically concerning dyspnea and fatigue, which are key symptoms prompting the indication of CCTA. These changes in clinical presentation can provide insight into the better rate of detection seen during the COVID-19 timeframe. An explanation could be represented by the repurposing of secondary-level cardiology units as COVID-19 or COVID-19 support wards—thus, patients with cardiovascular symptoms were more readily directed to tertiary care facilities. At the same time, these findings may be attributed to the emergence of new cases of dyspnea and other respiratory symptoms during the COVID-19 period, potentially of viral origin, suggesting a SARS-CoV-2 direct implication. It is conceivable that these symptoms, initially interpreted as indicative of cardiac involvement, prompted further investigation with CCTA. This underscores the complex interplay between symptomatology during COVID-19 and the clinical evaluation of patients with coronary artery anomalies.

During the COVID-19 pandemic, it became necessary to acknowledge the relationship between viral infections and pre-existing conditions, including CAAs and newly emerging cardiac conditions that may have been asymptomatic or mildly symptomatic before. Nevertheless, in our study, while the detection of group A anomalies was notable during the COVID-19 period (51.3%), they were not dominant. Being more severe in nature may indeed have led to their earlier discovery due to their symptomatic presentation.

On the other hand, our research identified a significant increase in the detection of anomalies from group B during the COVID-19 period, rising from 49% before the pandemic to 61.4%. This observed shift might have various reasons behind it. One possibility is that anomalies from group B, characterised by their potential to remain asymptomatic for extended periods or manifest later in life, were more frequently detected during the COVID-19 period due to heightened vigilance and increased utilisation of imaging techniques like CCTA. The decreased prevalence of STEMI during COVID-19 among these patients with CAAs might indicate a shift in the severity of cases presented for CCTA. This could suggest a potential loosening of the selection criteria and increased availability of the procedure. Patients presenting with COVID-19 manifestations may have undergone thorough diagnostic evaluations, including CCTA scans, leading to the incidental detection of previously undiagnosed anomalies from this group. At the same time, it is important to consider the potential role of the viral infection itself in precipitating the cardiac manifestations of these anomalies. The physiological stress induced by SARS-CoV-2 infection could exacerbate underlying cardiovascular issues, leading to a higher detection of CAAs during diagnostic procedures.

Regarding the dissemination of CAAs across genders, this study revealed a higher prevalence among males compared to women, similar to the findings reported by Barriales Villa et al. in their study. This occurrence contrasts with findings by Angelini et al. and Diez et al., who reported a higher prevalence in females [17]. However, it is important to note that the observed gender disparity in our study was not found to be statistically significant. Nevertheless, this observed disparity may be attributable to biological and genetic factors, potentially contributing to an elevated incidence of cardiovascular diseases in the male population of our region. It is noteworthy that these patterns could exhibit geographical variations, underscoring the importance of regional factors in influencing the detection of CAAs. Still, the consistent gender distribution among patients diagnosed with CAAs in our study across both evaluated time frames, despite numerical variations, suggests a robustness in the data integrity. Such stability in gender distribution strengthens the reliability of the study findings and underscores that any observed variations in CAA detection are less likely due to biases in patient selection or diagnostic practices.

When analysing age intervals, age ranges shifted during COVID-19, with patients slightly older on average (59.55 years) compared to before (57.61 years). The most substantial numbers of patients were observed between the ages of 50 and 70, both before and during the COVID-19 pandemic. This phenomenon may be attributed to the differing initial manifestations of heart disease between men and women at age 55; men commonly present with coronary artery disease, while women are more predisposed to heart failure as the first cardiac disorder [18]. Moreover, it is noteworthy to mention that older individuals were detected more frequently during the COVID-19 period, likely due to their increased vulnerability to the virus. The severity of clinical manifestations was heightened in older patients due to their frequently compromised immune function, rendering them susceptible to the impacts of COVID-19 [19].

While comparing the prevalence of common cardiovascular risk factors before and during the COVID-19 pandemic, our analysis revealed relatively similar percentages of patients with hypertension, diabetes mellitus, dyslipidemia, metabolic syndrome, and smoking history across both time periods. These findings suggest that there are no significant relevant statistical disparities in the prevalence of cardiovascular risk factors before and during COVID-19. However, it is essential to recognise that, while the pandemic may not have directly influenced the prevalence of these risk factors, the stability in their prevalence suggests a degree of resilience in healthcare delivery and patient behaviour despite the challenges posed by the pandemic.

The comparison of cardiovascular disorders prevalence before and during the COVID-19 pandemic suggests that during the pandemic, all three conditions showed a decrease; however, none of these changes demonstrated statistical significance.

CAC is a composite measure that considers both the extent and density of calcified lesions, providing a weighted evaluation of their presence in a given area [20]. CAC, used as an important and non-invasive instrument to assess the presence and degree of atherosclerosis, stands out as the foremost predictor for long-term, cause-specific mortality across various patient demographics, including age, ethnicity, sex, and risk factors [21,22]. In our study, during COVID-19, while the proportion of absent coronary artery calcium scores decreased slightly from 56.9 to 51.6%, there was relative stability in mild scores (26.1%). However, there was a noticeable increase in the moderate (14.9%) and severe (7.5%) categories during the pandemic period. This could suggest a potential worsening in coronary artery disease severity among patients evaluated during COVID-19 compared to before.

The COVID-19 pandemic is expected to substantially increase the burden and prevalence of cardiovascular diseases globally, posing long-term challenges for both patients and healthcare systems. This chronic impact is likely to have further consequences on life expectancy, highlighting the need for ongoing monitoring [23].

Upon analysis of patient data before and during the pandemic, we observed notable differences in the presentation of coronary artery anomalies. Our findings underscore the need to explore the potential influences of changes in screening practices, accessibility to medical services, and the direct impact of the pandemic on these variations. This highlights the need for further research into the complex interplay between medical resources, pandemic dynamics, and the manifestation of coronary anomalies.

## 5. Conclusions

Our research reveals a significant detection increase for CAAs by CCTA during the COVID-19 era, either highlighting a potential association between COVID-19 and cardiovascular symptoms emerging in susceptible individuals and/or underlining the role of primary/secondary level unit patient triage. Standardised clinical evaluation criteria employed for patient selection for CCTA probably have an effect on the CAA detection rate. Although established screening protocols are lacking, the utilisation of advanced imaging modalities like CCTA holds promise in increasing the capacity to identify and manage CAAs, not only during public health emergencies, but also in routine clinical practice.

## Figures and Tables

**Table 1:** Symptoms among patients with coronary artery anomalies before and during the COVID-19 pandemic (significant differences are presented in bold script).

Symptoms	All Cases (n = 350)		Before COVID-19 (n = 192)		During COVID-19 (n = 158)		*p* Values for Chi2 Test
	n	%	n	%	n	%	
Angina	190	54.3	112	58.3	78	49.4	0.058
Dyspnea	**145**	**41.4**	**70**	**36.5**	**75**	**47.5**	**0.037**
Syncope	11	3.1	7	3.6	4	2.5	0.391
Palpitations	109	31.1	59	30.7	50	31.6	0.472
Fatigue	**122**	**34.9**	**58**	**30.2**	**64**	**40.5**	**0.029**

**Table 2:** Electrocardiography changes among patients with coronary artery anomalies before and during the COVID-19 pandemic (significant differences are presented in bold script).

ECG	All Cases (n = 350)		Before COVID-19(n = 192)		During COVID-19 (n = 158)		*p* Values for Chi2 Test
	n	%	n	%	n	%	
Supraventricular tachycardia	88	25.1	55	28.6	33	20.9	0.061
Malignant ventricular dysrhythmias	**13**	**3.7**	**11**	**5.7**	**2**	**1.3**	**0.024**
Bradycardia	19	5.4	11	5.7	8	5.1	0.488
Atrioventricular block, first degree	15	4.3	5	2.6	10	6.3	0.074
Atrioventricular block, second degree	8	2.3	4	2.1	4	2.5	0.527
Atrioventricular block, third degree	12	3.4	5	2.6	7	4.4	0.260
Right bundle branch block	**15**	**4.3**	**12**	**6.3**	**3**	**1.9**	**0.038**
Left bundle branch block	17	4.9	8	4.2	9	5.7	0.338
Premature atrial complexes	21	6.0	9	4.7	12	7.6	0.180
Premature ventricular complexes	17	4.9	7	3.6	10	6.3	0.181
ST-elevation myocardial infarction (STEMI)	**36**	**10.3**	**28**	**14.6**	**8**	**5.1**	**0.002**
Non-STEMI	7	2.0	3	1.6	4	2.5	0.394

**Table 3:** Cardiovascular risk factors among patients with coronary artery anomalies before and during the COVID-19 pandemic.

Risk Factors	Before COVID-19 (n = 192)		During COVID-19 (n = 158)	
	n	%	n	%
Hypertension	87	45.3	62	39.2
Diabetes mellitus	24	12.5	20	12.7
Dyslipidemia	55	28.6	42	26.6
Metabolic syndrome	26	13.5	19	12
Smoking	28	14.6	17	10.8

**Table 4:** Coronary artery disease (CAD) and chronic cardiac failure among patients with CAAs before and during the COVID-19 pandemic.

Cardiovascular Disorder	Before COVID-19 (n = 192)		During COVID-19 (n = 158)	
	n	%	n	%
Coronary artery disease (CAD)	60	31.3	48	30.4
Chronic cardiac failure	71	37	50	31.6
Dilated cardiomyopathy (DCM)	31	16.1	16	10.1

## Data Availability

Data are contained within the article.
