# Correction: Zamfir et al. Trends in Coronary Artery Anomalies Detection by Coronary Computed Tomography Angiography (CCTA): A Real-Life Comparative Study before and during the COVID-19 Pandemic. *Healthcare* 2024, *12*, 1091

**DOI:** 10.3390/healthcare12161561

**Published:** 2024-08-07

**Authors:** Alexandra-Simona Zamfir, Tudor-Andrei Cernomaz, Bogdan Mihnea Ciuntu, Doina Azoicăi, Carmen Lăcrămioara Zamfir, Raluca Ozana Chistol, Anca Sava

**Affiliations:** 1 Clinical Hospital of Pulmonary Diseases, 700115 Iasi, Romania; 2 Department of Medical Sciences III, Faculty of Medicine, “Grigore T. Popa” University of Medicine and Pharmacy, 700115 Iasi, Romania; 3 Regional Institute of Oncology, 700483 Iasi, Romania; 4 Department of Surgery, “Grigore T. Popa” University of Medicine and Pharmacy, 700115 Iasi, Romania; 5 Department of Surgery, “St. Spiridon” County Clinical Emergency Hospital, 700111 Iasi, Romania; 6 Department of Preventive Medicine and Interdisciplinarity, “Grigore T. Popa” University of Medicine and Pharmacy, 700115 Iași, Romania; 7 Department of Morpho-Functional Sciences I, Faculty of Medicine, “Grigore T. Popa” University of Medicine and Pharmacy, 700115 Iasi, Romania; 8 Department of Medical Imaging, “Prof. Dr. George I.M. Georgescu” Cardiovascular Diseases Institute, 700503 Iași, Romania

## Error in Table

In the original publication [1], there was a mistake in Tables 3 and 4 as published. The values in Tables 3 and 4 were reported incorrectly—they actually represent the percentage of negative cases (mirror values). The corrected Table 3 and Table 4 appear below.

## Text Correction

There was an error in the original publication. Coronary heart disease was not the most common disorder; chronic cardiac failure was.

A correction has been made to **Results**, paragraph 9, as follows:

In terms of the associated comorbidities of these patients, cardiovascular conditions remained prevalent across both selected time frames, with chronic cardiac failure exhibiting the highest prevalence, followed by coronary artery disease (CAD) and dilated cardiomyopathy (Table 4).

There was an error in the original publication. The prevalence of hypertension during COVID-19 did not increase. At the same time, not only DCM showed a decrease, but all three conditions (DCM, coronary heart disease, and chronic heart failure).

A correction has been made to **Discussion**, paragraphs 10–11, as follows:

While comparing the prevalence of common cardiovascular risk factors before and during the COVID-19 pandemic, our analysis revealed relatively similar percentages of patients with hypertension, diabetes mellitus, dyslipidemia, metabolic syndrome, and smoking history across both time periods. These findings suggest that there are no significant relevant statistical disparities in the prevalence of cardiovascular risk factors before and during COVID-19. However, it is essential to recognise that, while the pandemic may not have directly influenced the prevalence of these risk factors, the stability in their prevalence suggests a degree of resilience in healthcare delivery and patient behaviour despite the challenges posed by the pandemic.

The comparison of cardiovascular disorders prevalence before and during the COVID-19 pandemic suggests that during the pandemic, all three conditions showed a decrease; however, none of these changes demonstrated statistical significance.

The authors state that the scientific conclusions are unaffected. This correction was approved by the Academic Editor. The original publication has also been updated.

## Figures and Tables

**Table 3:** Cardiovascular risk factors among patients with coronary artery anomalies before and during the COVID-19 pandemic.

Risk Factors	Before COVID-19 (n = 192)		During COVID-19 (n = 158)	
	n	%	n	%
Hypertension	87	45.3	62	39.2
Diabetes mellitus	24	12.5	20	12.7
Dyslipidemia	55	28.6	42	26.6
Metabolic syndrome	26	13.5	19	12
Smoking	28	14.6	17	10.8

**Table 4:** Coronary artery disease (CAD) and chronic cardiac failure among patients with CAAs before and during the COVID-19 pandemic.

Cardiovascular Disorder	Before COVID-19 (n = 192)		During COVID-19 (n = 158)	
	n	%	n	%
Coronary artery disease (CAD)	60	31.3	48	30.4
Chronic cardiac failure	71	37	50	31.6
Dilated cardiomyopathy (DCM)	31	16.1	16	10.1
